# Draft genome sequence of *Solidesulfovibrio* sp. strain C21 isolated from Laguna Grande (Lagunas de Villafranca), Spain

**DOI:** 10.1128/mra.00165-25

**Published:** 2025-05-20

**Authors:** Velislava Ilieva, Felipe Gomez, Daniel Read, Karen Olsson-Francis, Michael C. Macey

**Affiliations:** 1 AstrobiologyOU, School of Environment, Earth and Ecosystem Sciences, The Open University 5488, Milton Keynes, United Kingdom; 2 Centro de Astrobiologia (CSIC-INTA) 120422 , Torrejón de Ardoz, Spain; 3 Centre for Ecology and Hydrology, Wallingford, United Kingdom; DOE Joint Genome Institute, Berkeley, California, USA

**Keywords:** extremophiles, sulfate reduction, halophiles, Spain

## Abstract

Here, we report the draft genome sequence of a strain of *Solidesulfovibrio* isolated from hypersaline sediment samples collected from Laguna Grande (Lagunas de Villafranca), La Mancha Region, Spain. This genome sequence enables further investigation into the genetic and metabolic diversity of sulfate reducers in extreme, hypersaline environments.

## ANNOUNCEMENT

Laguna Grande is in the wetland complex Lagunas de Villafranca in the La Mancha region of Spain. It is a perennial alkaline (pH 9), saline (~10%–15% salinity) and sulfate-rich lake. The high sulfate concentration of the lake water is influenced by the leaching of materials from the Triassic, including gypsum marls, sandstones, and limestones (1). Previous studies of other lakes in the La Mancha region have identified a dominance of halophilic bacteria and archaea and the presence of sulfate-reducing bacteria (2–4). Here, we present the draft genome of *Solidesulfovibrio* sp. strain C21 isolated from Laguna Grande. The strain was isolated from sediment samples collected from a depth of 5 cm on 9 November 2022 (39°27′13.2″N 3°20′18.3″W).

Sedment samples were enriched with Postgate Medium containing 4 M NaCl (5). Serial dilutions were prepared and plated onto solid Postgate Medium and incubated at 30°C for 30 days. Distinct colonies were observed, leading to the isolation of *Solidesulfovibrio* sp. strain C21. This strain was selected for genomic characterization to expand our knowledge of sulfur cycling in extreme environments. Pure colonies were used to establish cultures. To extract DNA, cell suspensions were lysed with TE buffer containing lysozyme (MPBio), metapolyzyme (Sigma-Aldrich), and RNase A (ITW Reagents), incubated for 25 min at 37°C. Proteinase K (VWR Chemicals, 0.1 mg/mL) and SDS (Sigma-Aldrich, 0.5% v/v) were added and incubated for 5 min at 65°C. Genomic DNA was purified using an equal volume of SPRI beads and resuspended in 10 mM Tris-HCl (pH 8.0). DNA was quantified with the Quant-iT dsDNA HS assay (Thermo Fisher Scientific) using an Eppendorf AF2200 plate reader and diluted as needed. DNA libraries were prepared by MicrobesNG (Birmingham, United Kingdom) using the Nextera XT Library Prep Kit (Illumina, USA). This was carried out according to the manufacturer’s instructions, with the exceptions of a twofold increase in DNA input and a 45 s PCR elongation time. DNA quantification and library preparations were carried out on a Hamilton Microlab STAR automated liquid handling system (Hamilton Bonaduz AG, Switzerland). Libraries were sequenced on an Illumina NovaSeq 6000 (Illumina, San Diego, USA) using a 250 bp paired-end protocol.

A total of 2,981,332 reads were trimmed using Trimmomatic (v0.30; default settings) (6), and assembly was carried out with SPAdes (v3.7; default settings) (7). Genome quality was assessed with CheckM (v1.4.0; default settings) (8), coverage calculated using BWA, SAMtools (0.1.19), and BEDTools genomecov (2.2.7) (9–11). Taxonomy of the assembled genome was investigated *via* Protologger and the Type Strain Genomic Server (12, 13). Genome annotation was performed using the NCBI prokaryotic genome annotation pipeline (v1.2) (14), and metabolic pathways assessed using BlastKoala (2.3) and DRAM (v0.1.2) (15, 16).

*Solidesulfovibrio* sp. strain C21 was most closely related to *Solidesulfovibrio marrakechensis* (99.29% 16S rRNA gene sequence identity) determined by Protologger. ANI and dDDH scores identified the genome as novel, matching best to *Solidesulfovibrio fructosivorans* (92% and 70%, respectively). The genome size was 4,501,810 bp with 84-fold coverage, 63.40% GC content, and an *N*_50_ value of 51,199 bp. The assembled genome is 169 contigs with 3,983 coding sequences, 48 tRNAs, and one copy of 5S, 16S and 23S rRNA. CheckM identified completion and contamination values as 99.4% and 0%, respectively. The genome contained genes encoding enzymes for dissimilatory sulfate reduction (*sat*, *aprAB*, and *dsrAB*), nitrate reduction (*napAB* and *nrfAH*), nitrogen fixation (*nifDKH*), and urea hydrolysis (*ureABC*).

## Data Availability

This Whole Genome Shotgun project has been deposited at DDBJ/ENA/GenBank under the accession JBLODY000000000. The version described in this paper is version JBLODY010000000. Raw reads are available at SRR32488972.
